# *In vitro* and *in vivo* evaluation of the effects of condensed tannins and catechins monomers on antioxidant and intestinal health of Chinese seabass (*Lateolabrax maculatus*)

**DOI:** 10.3389/fvets.2025.1558942

**Published:** 2025-02-25

**Authors:** Ruiqi Dong, Jianqiang Qiu, Junming Cao, Wen Huang, Bing Chen, Hongxia Zhao, Wenhao Sun, Huijie Lu, Jiun-Yan Loh, Kai Peng

**Affiliations:** ^1^Key Laboratory of Animal Nutrition and Feed Science in South China, Ministry of Agriculture and Rural Affairs, Guangdong Provincial Key Laboratory of Animal Breeding and Nutrition, Collaborative Innovation Center of Aquatic Sciences, Institute of Animal Science, Guangdong Academy of Agricultural Sciences, Guangzhou, China; ^2^College of Fisheries and Life Sciences, Shanghai Ocean University, Shanghai, China; ^3^College of Fisheries, Huazhong Agricultural University, Wuhan, China; ^4^College of Fisheries, Guangdong Ocean University, Zhanjiang, China; ^5^Tropical Futures Institute, James Cook University, Singapore, Singapore

**Keywords:** condensed tannins, catechins monomers, *Lateolabrax maculatus*, antioxidant, intestinal health

## Abstract

Plant-derived condensed tannins (CT) exhibit strong bioactivity of antioxidant, immunostimulation and intestinal protection, but with little clues of the mechanism of action. Since CT are consist of catechins (CAs) monomers, e.g., catechin (CA), epicatechin (EC) and epigallocatechin (EG), we motivated to use the monomers to explore the underlying mechanisms in a seabass model focusing on anti-oxidative stress and intestinal health of *Lateolabrax maculatus*. An *in vitro* intestinal primary cell oxidative stress model induced by hydrogen peroxide was set up to assess the antioxidant and immune activities of CT and CAs. Another 56–d feeding trial with 800 fish was conducted to evaluate the effects of CT and CAs on growth performance, intestinal permeability and digestive enzyme activities, intestinal morphology and antioxidant status, and intestinal bacterial flora of fish. Five diets were prepared to contain 0 (G1) and 1 g/kg of CT, CA, EC and EG. Fish were randomly distributed into 20 tanks with 4 tanks per diet and 40 fish per tank, and were fed to apparent satiation twice daily. Results showed that CT and CAs exhibited similar effects in alleviating hydrogen peroxide-induced cell injury by activating nuclear factor erythroid 2-related factor 2 gene expression, and improving antioxidant and immune capacities. Dietary CT and CAs enhanced intestinal antioxidant ability and increased (*p* < 0.05) the abundance of intestinal Firmicutes, Proteobacteria and Bacteroidetes to oxidative stress tolerant. With a dose of 1 g/kg CT and CA promoted (*p* < 0.05) intestinal total antioxidant capacity, but slightly induced intestinal injury mainly due to increased (*p* < 0.05) intestinal permeability (as reflected by increased lipopolysaccharide concentrations) and inhibited (*p* < 0.05) digestion (as reflected by the decreased trypsin and lipase activities) of fish. In summary, CT and CAs protect intestine from oxidative stress and improve intestinal antioxidant capacity by stimulating antioxidant enzyme system and bacterial flora. CA and EC show similar or superior antioxidant activity than CT.

## Graphical abstract

Schematic model showing a summary of the study findings.

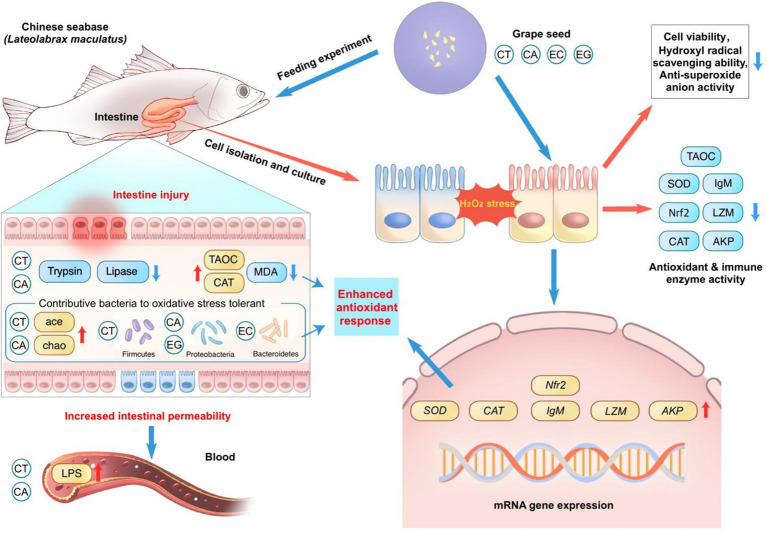

## 1 Introduction

The prohibition of antibiotics has posed huge challenges to both feed and food-producing animal industries. There is an urgent need to explore antibiotic alternative products and technologies. Plant extracts are considered as promising antibiotic substitutes due to their growth-promoting, antioxidant and immunopotentiation effects (1). A good example is about condensed tannins (CT) that are natural plant polyphenolic compounds with evident potent antioxidant and stress-resistant effects showed in various animal models (2). Our previous studies showed that low dose CT promoted growth performance of shrimp (3) and improved intestinal health of fish (4). Furthermore, CT could alleviate oxidative damage by regulating intestinal bacterial microbiota of Chinese sea bass (*Lateolabrax maculatus*) (5), and increase the intestinal cell vitality and antioxidant capacity of fish *in vitro* by inhibiting the production of reactive oxygen species (ROS) (6).

It is commonly regarded that the biological activity of CT is closely related to their chemical structure (7). As a polyphenol polymer, CT are mainly composed of three catechins (CAs) monomers, i.e., catechin (CA), epicatechin (EC) and epigallocatechin (EG) (8). Déprez et al. (9) documented that polymeric proanthocyanidins (i.e., CT) were catabolized by human intestinal microflora into low-molecular-weight phenolic acids starting from depolymerization of polymer to CAs monomers (i.e., CA and EC). CAs are the critical compounds responsible for the claimed health benefits of polyphenols, including antioxidant and anti-inflammatory activities (10). For instance, CA was found to reduce hepatic damage by suppressing oxidative stress and controlling the transcription factor expression involved in stellate cell activation (11). EC was reported to prevent myocardial infarction in rats due to its antioxidant, anti-inflammatory, anti-tachycardial, and anti-cardiac hypertrophic effects (12). EG was documented to prevents oxidative stress-induced cellular senescence in human mesenchymal stem cells via activating the nuclear factor erythroid 2-related factor 2 (Nrf2) signaling pathway (13). Although the anti-oxidative stress activity and feeding value of CT on fish have been evaluated (6), information about CAs is rare. It was reported that CA from green tea had the potential to decrease the chlorpyrifos-induced oxidative stress in larval zebrafish (*Danio rerio*) (14). Dietary EC at 1 g/kg was reported to enhance muscle antioxidant capacity, improve fillet quality, and promote myofiber development in yellow river carp (*Cyprinus carpio*) (15). Inclusion of EG (epigallocatechin-3-gallate) in Chinese rice field eel (*Monopterus albus*) diets improved nonspecific immune response (16). Our previous studies demonstrated that CT relieve oxidative stress of *L. maculatus* by activating the Nrf2 signaling pathway (6, 17), which plays a significant role in protecting cells from oxidative stresses (18). Also, CAs including CA, EC and EG or their esterified form can induce the activation of Nrf2 signaling pathway (19–21). Understanding bioactivity differences between CT and CAs would help elucidate the antioxidative mechanism of CT.

It is well-known that intestine is the largest digestive and immune organ in fish, so the intestinal health is essential for maintaining normal growth and stress resistance of fish. However, there is scarce information about the effects of CT and their monomers on intestinal health. The objectives of this study were to assess the effects of CT and CAs on the survival and antioxidant and immune capacities of intestinal mucosal cells in *L. maculatus* and to evaluate the effects of dietary CT and CAs on growth performance, intestinal permeability and digestive enzyme activities, intestinal morphology and antioxidant status, and intestinal bacterial flora of fish.

## 2 Materials and methods

The protocol (no. GDAAS2022666) and all procedures of the experiment were approved by the Animal Care and Use Committee of the Collaborative Innovation Center of Aquatic Sciences, Guangdong Academy of Agricultural Sciences (Guangzhou, China).

### 2.1 *In vitro* experiment

#### 2.1.1 Cell isolation, culture and treatment

A total of fifty *L. maculatus* (approximately 500 g per fish) were provided by Jianyi hatchery (Zhuhai, China). The intestinal mucosal cells of fish were isolated according to the procedures described by Peng et al. (6). Briefly, fish were fasted for 24 h before sampling. A total of 10 fish were collected and dissected to obtain the intestines. The intestinal mucosa was separated, pooled and rinsed with 20 mL D-Hanks solution (Sigma-Aldrich, H6648) for 4 times using 10 mL injection syringe. The D-Hanks solution contains 0.1% of penicillin–streptomycin-gentamicin solution (Beyotime, C0223) to protect cells from microbial contamination. The intestinal mucosa was treated with collagenase I and IV (Sigma) at 28°C for 0.5 h and centrifuged at 15.0 × *g* for 5 min to obtain the cell mass. The cell mass was cultured (28°C with 6% of carbon dioxide) in 96-well culture plate (approximately 2.2 × 10^3^ cell per well) at the cell incubator for further treatment.

After 24 h incubation, cells were assigned to 6 groups, i.e., normal control (NC), hydrogen peroxide (HP) (Sigma-Aldrich, H6520), CT, CA, EC and EG, with 12 replicates (wells) in each group. The grape seed CT was extracted and purified according to the method as described by Peng et al. (6). Purified CAs monomers (derived from grape seed) were obtained from the Shanghai Yuanye Biotechnology Co., Ltd. (Shanghai, China). Cells in the NC group were incubated with the minimum essential medium (MEM) (Gibco, United States) containing 100 μmol/L of sterile normal saline. Cells in other groups were incubated with the MEM containing 100 μmol/L of hydrogen peroxide solution. After 12 h of incubation, cells in the CT, CA, EC and EG groups were treated with 800 μmol/L of condensed tannins, catechin, epicatechin and epigallocatechin, respectively. Incubation concentrations of HP, CT and CAs were selected depending on relevant pre-experiment. Cells in the NC and HP groups were treated with 800 μmol/L of sterile normal saline. Cell culture in all groups were terminated after 24 h of incubation. The cell-free medium in the wells of culture plate was removed by pipette and the wells were rinsed using the PBS solution (Gibco, C10010500BT, 0.01 mol/L). Cells were harvested by incubation of the whole cell culture in the 0.25% of trypsin–EDTA solution (Gibco, 25200-056) for 10 min followed by adding M199 culture medium containing 15% of fetal bovine serum (Gibco, United States), and centrifuged at 1200 × *g* for 5 min. The obtained cell fraction was divided into two portions. One portion was used to determine the cell viability, hydroxyl radical scavenging ability, and anti-superoxide anion activity. Another portion was stored at −80°C for subsequent analysis of cell antioxidant and immune capacity.

#### 2.1.2 Determinations of cell viability, hydroxyl radical scavenging ability, and anti-superoxide anion activity

The cell viability assay kit (CellTiter-Glo^®^, Promega, United States) was used to determine the cell viability by the luminescent method. Briefly, remove the cell culture plate and balance at room temperature for 10 min, add 100 μL CellTiter-Lumi™ luminescence detection reagent to each hole of 96-well plate (1,500 cells per well), and oscillate at room temperature for 2 min to promote cell lysis, then incubate at room temperature for 10 min to stabilize the luminous signal followed by chemiluminescence detection using a multifunctional enzyme marker. The relative cell viability is calculated directly from the chemiluminescence readings following manufacturer’s instructions.

The cell hydroxyl radical scavenging ability was measured by using commercial kit (A004-96 T) provided by the Shanghai HuicH Biotechnology Co., Ltd. (Shanghai, China). Briefly, remove the cell culture plate and balance at room temperature for 10 min, transfer 10^6^ cells into a 1.5 mL centrifuge tube and add 500 μL extraction buffer (included in the commercial kit) into the tube for cell lysis, then centrifuge at 15000 × *g* for 10 min (4°C) and collect the supernatant. Transfer the reagent (included in the commercial kit) into 200 μL supernatant and incubate the reaction mixture at 37°C for 30 min. The incubation was mixed with 40 μL 2-thiobarbituric acid, put into boiling water for 15 min and then let cool at room temperature for 20 min. The cell hydroxyl radical scavenging ability of mixture was calculated from the absorption value at 532 nm following manufacturer’s instructions.

The inhibition and produce superoxide anion assay kit (A052-1-1) obtained from the Nanjing Jiancheng Bioengineering Institute (Nanjing, China) was used to analyze the cell anti-superoxide anion activity. Briefly, remove the cell culture plate and balance at room temperature for 10 min. The cells were collected, cleaned with 1 mL PBS for 2 times and centrifuged at 1000 × *g* to collect the precipitated cells. Then 0.5 mL PBS buffer (0.1 mol/L, pH 7.4) was added to suspend the cells. The cells were crushed by ultrasound, and the broken cell suspension was taken to be measured following manufacturer’s instructions.

#### 2.1.3 Determinations of cell antioxidant and immune capacity

Commercial kits (Nanjing Jiancheng Bioengineering Institute, Nanjing, China) were used to determine the cell antioxidant and immune indices following manufacturer’s instructions, including total antioxidant capacity (TAOC, A015-1-2), catalase (CAT, A007-1-1), superoxide dismutase (SOD, A001-1-2), glutathione peroxidase (GPx, A005-1-2), alkline phosphatase (AKP, A059-2-2), immunoglobulin M (IgM, E025-1-1) and lysozyme (LZM, A050-1-1). The mRNA expression of *Nrf2*, *CAT*, *SOD*, *IgM*, *LZM* and *AKP* were determined using the real-time PCR analysis and calculated according to the 2^−∆∆Ct^ method (22). Primers sequences and the real-time PCR cycling conditions were shown in Table 1.

**Table 1:** Primers used for real-time PCR.

Gene	Forward (5′ to 3′)	Reverse (3′ to 5′)	Accession number	PCR cycling conditions
*Nrf2*	AACTAACGTGAACGGGCTCC	GGTTCATGTCCTGTTGACTGC	KU892416.1	5 min at 95°C, followed by 40 cycles of 10 s at 95°C, 34 s at 60°C and 30 s at 72°C.
*CAT*	ACAGCTTCAGTGCTCCTGAC	TTTGCACCATGCGTTTCTGG	XM_021470442.1	
*SOD*	TTTACGACCTGTCCGCGAAA	TTTACGACCTGTCCGCGAAA	NM_131294.1	
*IgM*	GTGGACGGGTGGTAAAGTGT	AGCAAGACGCCACATACACA	MN866897.1	
*LZM*	GCAGCCATCCATTTAGAAGGTAG	GTAGCGCCAGATTCGGTGG	NM_139180.1	
*AKP*	CGTCAGGCTCAAGAAGCTCA	CGTTTGGAGACGTGACAGGA	AY144372.1	
*β-actin*	GTGAAATTCTTGGACCGGCG	GGATCGCTAGTTGGCATCGT	AB089346.1	

Nrf2, nuclear factor erythroid 2-related factor 2; CAT, catalase; SOD, superoxide dismutase; IgM, immunoglobulin M; LZM, lysozyme; AKP, alkline phosphatase; β-actin, internal reference gene.

### 2.2 *In vivo* experiment

#### 2.2.1 Diets preparation, experimental design and feeding management

The diet formula was shown in Table 2. Five diets were prepared to contain 0 (G1) and 1 g/kg of CT (G2), CA (G3), EC (G4) or EG (G5). Dietary concentration of CT was selected according to the previous study by Peng et al. (23). All ingredients were well mixed, extruded into 2 mm pellets, dried in oven at 55°C for 12 h, and stored in sealed plastic bags at −20°C prior to use. Actual concentrations of CT, CA, EC and EG in the G1, G2, G3, G4 and G5 were 0, 1.2, 1.0, 1.1 and 1.0 g/kg, which were determined by the reversed-phase high performance liquid chromatography method as described by Chen et al. (24).

**Table 2:** Ingredients and nutrient compositions (g/kg DM) of experimental diets.

Ingredients	Diets				
	G1	G2	G3	G4	G5
Fish meal	300	300	300	300	300
Casein	140	140	140	140	140
Soy protein concentrate	70	70	70	70	70
Wheat flour	200	200	200	200	200
Monocalcium phosphate	15	15	15	15	15
Fish oil	40	40	40	40	40
Soybean oil	20	20	20	20	20
Soy lecithin	20	20	20	20	20
Vitamin premix	1	1	1	1	1
Mineral premix	5	5	5	5	5
Choline chloride	5	5	5	5	5
Cellulose	184	183	183	183	183
Condensed tannins	0	1	0	0	0
Catechin	0	0	1	0	0
Epicatechin	0	0	0	1	0
Epigallocatechin	0	0	0	0	1
Total	1,000	1,000	1,000	1,000	1,000
Analyzed nutrients compositions					
Dry matter	950	944	942	943	948
Crude protein	396	392	398	397	400
Ether extract	98	95	96	95	97
Ash	74	72	73	72	73

G1, basal diet; G2, basal diet supplemented with 1 g/kg of condensed tannins; G3, basal diet supplemented with 1 g/kg of catechin; G4, basal diet supplemented with 1 g/kg of epicatechin; G5, basal diet supplemented with 1 g/kg of epigallocatechin. One kilogram of diet provides: Vitamin A, 3,230 IU; Vitamin D_3_, 1,600 IU; Vitamin E, 160 mg; Vitamin K_3_, 4 mg; Vitamin B_1_, 4 mg; Vitamin B_2_, 8 mg; Vitamin B_6_, 4.8 mg; Vitamin B_12_, 0.016 mg; nicotinic acid, 28 mg; pantothenic acid calcium, 16 mg; biotin, 0.064 mg; folic acid, 1.285 mg; inositol, 40 mg; Ca, 1,150 mg; K, 180 mg; Mg, 45 mg; Fe, 50 mg; Zn, 40 mg; Mn, 9.5 mg; Cu, 7.5 mg; Co, 1.25 mg; I, 0.16 mg; Se, 0.25 mg; lysine, 28.5 g; methionine, 10.9 g; leucine, 32.1 g; isoleucine, 18.2 g; phenylalanine, 18.2 g; valine, 21.6 g; tyrosine, 16.1 g; alanine, 21.1 g; arginine, 17.0 g; threonine, 16.6 g; glycine, 16.7 g; histidine, 8.0 g; serine, 18.1 g; glutamic acid, 74.6 g; aspartic acid, 32.9 g.

Fish were stocked in a cement pond (2.0 × 3.0 × 2.0 m) to acclimate to diets and condition for 2 weeks prior to start of the experiment. At the beginning of the 56–d feeding trial, a total of 800 fish with the initial body weight of 4.38 ± 0.02 g were randomly assigned to 20 tanks with 4 tanks per diet and 40 fish per tank. Fish were hand-fed to apparent satiation twice daily (08:30 and 18:30). Feces and uneaten feed were siphoned out before every feeding. Uneaten feed was collected in 0.5 h after each meal and dried at 55°C to a constant weight to calculate the feed intake (FI). During the feeding trial, water temperature 26.5–28.5°C, salinity 4–6 ‰, dissolved oxygen 6.0–8.0 mg/L, pH 7.8–8.1, ammonia nitrogen and nitrite concentrations 0.02–0.06 mg/L.

#### 2.2.2 Sampling and processing

At the end of the feeding trial, all fish were fasted for 24 h and anesthetized with 40 mg/L of the 3-aminobenzoic acid ethyl ester methanesulfonate (Sigma-Aldrich, United States) prior to sampling. Fish per tank were counted and weighted to calculate for the survival rate (SR), final body weight (FBW), weight gain rate (WGR), specific growth rate (SGR) and feed conversion rate (FCR). Six fish per tank were randomly picked out and dissected to collect the intestines to calculate the intestinesomatic index (ISI) (*n* = 4).

Blood was withdrawn from the caudal vein of 10 fish per tank using sterile syringe, pooled and incorporated into a 15 mL centrifuge tube, kept at 4°C for 2 h and then centrifuged at 1600 × *g* for 10 min. Subsequent serum was stored at −20°C for the analyses of serum metabolites (*n* = 4) of diamine oxidase, lipopolysaccharide and D-lactate. After blood collection, the intestines of these 10 fish were collected on ice, homogenized in ice-cold physiological saline solution, and centrifuged at 1200 × *g* for 15 min at 4°C. Supernatant was immediately stored at −20°C for analysis of trypsin, lipase and amylase (*n* = 4).

The intestines of 6 fish per tank were randomly collected for histological examination (*n* = 4). Intestines were fixed immediately in the 4% paraformaldehyde solution for 24 h. The fixed samples were dehydrated in the ethanol and treated with xylene, embedded in paraffin wax and sectioned in 5 μm slices, and then stained with hematoxylin-eosin (H&E) using standard histological techniques. Slices were examined under a light microscope (CX31, Olympus, Japan) and electronic images were obtained by a CCD camera (CV5000, Keyence, China).

Another 12 fish per tank were randomly selected to separate intestines. Intestine samples from 6 fish were transferred into a centrifuge tube and immediately stored at −20°C for subsequent determination of the intestinal antioxidant indices (*n* = 4), i.e., TAOC, CAT and SOD activities and the malonaldehyde (MDA) concentration. Another 6 intestine samples were collected to separate the intestinal contents and stored at −80°C for subsequent characterization of intestinal bacterial microbiome (*n* = 4).

#### 2.2.3 Determination of feed nutrients and serum metabolites levels, and intestinal digestive enzyme activities

The experimental diets were analyzed for dry matter (method 930.15), crude protein (method 984.13), ether extract (method 920.39) and ash (method 942.05) using the methods of AOAC (25).

Commercial kits from the Nanjing Jiancheng Bioengineering Institute (Nanjing, China) were used according to the methods described in other studies to determine the serum diamine oxidase (A088-2-1) activity (26) and lipopolysaccharide (H255-1-1) concentrations (27), and the intestinal trypsin (A080-2-2), lipase (A054-1-1) and amylase (C016-1-1) activities (28), as well as the TAOC, CAT, SOD activities and the MDA (A003-1-2) concentration (29). The serum D-lactate concentration was measured by the method described by Chen et al. (30) using the ELISA kit (600006A) provided by the Jiangsu Meibiao Biotechnology Co., Ltd. (Jiangsu, China).

#### 2.2.4 Bacterial DNA extraction and 16S rRNA sequencing

The bacterial DNA extraction and 16S rRNA sequencing were conducted following the procedures described by Peng et al. (31). In brief, bacterial DNA was extracted using the QIAamp DNA Stool Mini Kit (51,104, Qiagen, Germany). The concentration and purity of the genomic DNA were measured using a NanoDrop™ 2000c spectrophotometer (Thermo Scientific, United States). The DNA samples extracted from the intestines of the fish in each tank were combined to a sample and stored at −20°C for further analysis (32). Primers 515F (5’-GTGCCAGCMGCCGCGGTAA-3′) and 806R (5’-GGACTACHVGGGTWTCTAAT-3′) were used to target the V4 region of 16S rRNA genes (420 bp). The PCR was operated in duplicate with a 20 μL reaction volume containing 1 to 10 ng of DNA template, 0.3 μmol/L of each respective primer, and 10 μL of SsoAdvanced Universal SYBR Green supermix (Bio-Rad Laboratories, Inc., Hercules, CA, United States). The reaction conditions of PCR included initial denaturing step of 1 min at 98°C, followed by 30 cycles at 98°C for 10 s, 30 s at 50°C and 30 s at 72°C, and final extension of 5 min at 72°C. The 16S rRNA sequencing and library construction were conducted by the Guangzhou Xingyu Biotechnology Co., Ltd. (Guangzhou, China) with project no. YYR2023011101G. Sequencing was performed using an Illumina MiSeq platform (Illumina, United States) according to the standard protocols.

#### 2.2.5 Bioinformatics analysis

Sequencing reads were analyzed using the QIIME software package (v. 1.9.1). The processed reads were then clustered to the OTUs by the USEARCH software (v. 11.0.1) based on 97% sequence similarity. Taxonomic assignment was conducted using the Ribosomal Database Project Classifier (v. 2.12) based on the GreenGene 16S rRNA gene database (v. 13.8). Alpha diversity indexes such as ace, chao, shannon and simpson were calculated using the Phyloseq Package (v. 3.5.3) and compared using the Wilcoxon rank sum test on the R statistical computing platform. Beta diversity analysis was performed using R pheatmap package based on the Weighted-Unifrac distance matrix. The phenotypic contribution of bacterial phyla to oxidative stress tolerant was analyzed using the Bugbase phenotypic classification prediction analysis based on the Greengene database.

### 2.3 Calculations and statistical analysis

The SR, WGR, SGR, FI, FCR and ISI were calculated according to the equations described by Amoah et al. (33). All data were subjected to a one-way ANOVA using the SPSS 17.0 statistical software followed by the Duncan’s multiple-range test when the data met the homogeneity of variance. If the homogeneity of variance was not satisfied, the Dunnett’s T3 test method was utilized for multiple comparisons. The treatment in the cell experiment and the tank in the feeding trial were used as the statistical unit, respectively. Results were presented as the mean ± standard error. The level of significance was set at *p* < 0.05.

## 3 Results

### 3.1 Cell viability, hydroxyl radical scavenging ability, and anti-superoxide anion activity

**Figure 1:**
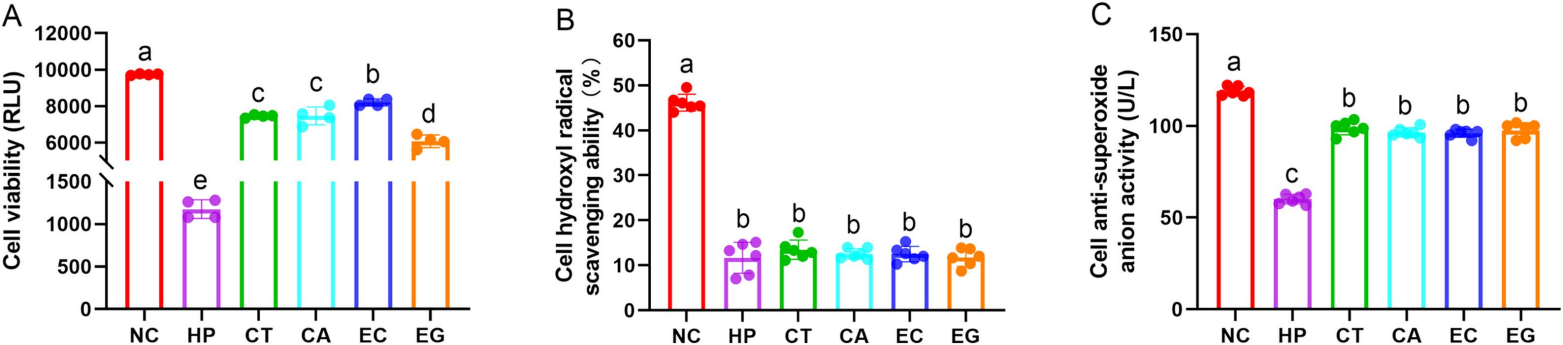
Cell viability **(A)**, hydroxyl radical scavenging ability **(B)**, and anti-superoxide anion activity **(C)** among treatments. NC, normal control; HP, cell treated with 100 μmol/L hydrogen peroxide; CT, cell treated with 100 μmol/L hydrogen peroxide and 800 μmol/L condensed tannins; CA, cell treated with 100 μmol/L hydrogen peroxide and 800 μmol/L catechin; EC, cell treated with 100 μmol/L hydrogen peroxide and 800 μmol/L epicatechin; EG, cell treated with 100 μmol/L hydrogen peroxide and 800 μmol/L epigallocatechin. Different lowercase letters above the bars denote significant differences among treatments from each other (*p* < 0.05).

Compared to NC, cell viability, hydroxyl radical scavenging ability, and anti-superoxide anion activity were decreased (*p* < 0.05) in HP (Figure 1). Cell viability and anti-superoxide anion activity in CT, CA, EC and EG were higher (*p* < 0.05) in HP but were lower (*p* < 0.05) than those in NC. The cell hydroxyl radical scavenging ability and cell anti-superoxide anion activity were similar (*p* > 0.05) among CT, CA, EC and EG. The CT and CA had similar (*p* > 0.05) cell viability. Compared to CT and CA, the cell viability was increased (*p* < 0.05) in EC but was decreased (*p* < 0.05) in EG.

### 3.2 Cell antioxidant and immune response

The cell antioxidant and immune indices were lower (*p* < 0.05) in HP than NC, but were higher (*p* < 0.05) in CT, CA, EC and EG than HP (Table 3). Compared to NC, the TAOC was decreased (*p* < 0.05) in CT and EG, the CAT and GPx were decreased (*p* < 0.05) in CT, CA, EC and EG, the SOD was decreased (*p* < 0.05) in CT and EG, the AKP was decreased (*p* < 0.05) but IgM was increased (*p* < 0.05) in CA, EC and EG. There was no difference (*p* > 0.05) in LZM among CT, CA, EC and EG.

**Table 3:** Cell antioxidant and immune response among treatments.

Items	Treatments					
	NC	HP	CT	CA	EC	EG
Antioxidant						
TAOC (mmol/L)	2.11 ± 0.03^a^	0.42 ± 0.01^d^	1.72 ± 0.12^b^	2.18 ± 0.03^a^	2.06 ± 0.09^a^	0.80 ± 0.03^c^
CAT (U/mL)	13.94 ± 0.32^a^	4.00 ± 0.06^c^	9.86 ± 0.11^b^	9.83 ± 0.11^b^	10.43 ± 0.26^b^	9.96 ± 0.17^b^
SOD (U/mL)	65.32 ± 0.91^ab^	10.56 ± 1.14^d^	60.94 ± 0.33^bc^	67.66 ± 2.27^a^	65.15 ± 1.31^ab^	60.31 ± 2.50^c^
GPx (U/mL)	110.37 ± 3.81^a^	30.77 ± 6.05^c^	80.68 ± 0.90^b^	78.98 ± 0.90^b^	71.52 ± 5.88^b^	72.88 ± 3.34^b^
Immune						
AKP (U/L)	16.63 ± 0.09^a^	8.24 ± 0.06^d^	16.52 ± 0.47^a^	13.80 ± 0.16^b^	13.85 ± 0.28^b^	12.14 ± 0.77^c^
IgM (μg/mL)	24.28 ± 0.96^bc^	13.90 ± 0.10^d^	23.17 ± 3.29^c^	28.73 ± 0.26^a^	27.51 ± 1.91^ab^	27.94 ± 1.05^ab^
LZM (U/mL)	28.13 ± 0.94^a^	11.57 ± 0.44^b^	28.74 ± 4.38^a^	28.53 ± 3.51^a^	28.18 ± 2.83^a^	28.37 ± 2.80^a^

NC, normal control; HP, cell treated with 100 μmol/L hydrogen peroxide; CT, cell treated with 100 μmol/L hydrogen peroxide and 800 μmol/L condensed tannins; CA, cell treated with 100 μmol/L hydrogen peroxide and 800 μmol/L catechin; EC, cell treated with 100 μmol/L hydrogen peroxide and 800 μmol/L epicatechin; EG, cell treated with 100 μmol/L hydrogen peroxide and 800 μmol/L epigallocatechin. TAOC, total antioxidant capacity; CAT, catalase; SOD, superoxide dismutase; GPx, glutathione peroxidase; AKP, alkline phosphatase; IgM, immunoglobulin M; LZM, lysozyme. Different superscript lowercase letters within a row indicate a significant difference among treatments from each other (*p* < 0.05).

**Figure 2:**
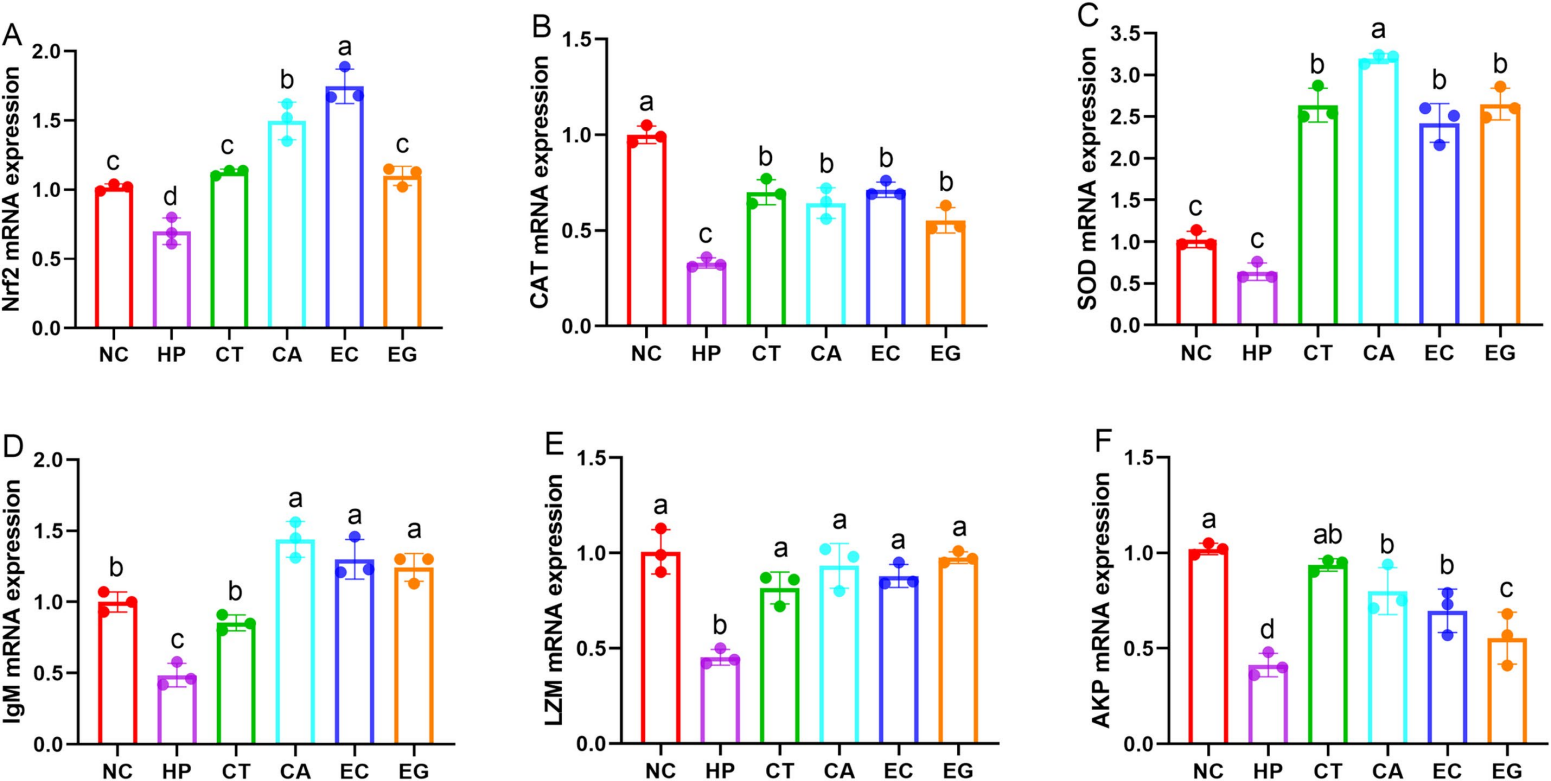
Cell gene expression of antioxidant and immune indices among treatments. *Nrf2*, nuclear factor erythroid 2-related factor 2 **(A)**; *CAT*, catalase **(B)**; *SOD*, superoxide dismutase **(C)**; *IgM*, immunoglobulin M **(D)**; *LZM*, lysozyme **(E)**; *AKP*, alkline phosphatase **(F)**; NC, normal control; HP, cell treated with 100 μmol/L hydrogen peroxide; CT, cell treated with 100 μmol/L hydrogen peroxide and 800 μmol/L condensed tannins; CA, cell treated with 100 μmol/L hydrogen peroxide and 800 μmol/L catechin; EC, cell treated with 100 μmol/L hydrogen peroxide and 800 μmol/L epicatechin; EG, cell treated with 100 μmol/L hydrogen peroxide and 800 μmol/L epigallocatechin. Different lowercase letters above the bars denote significant differences among treatments from each other (*p* < 0.05).

Compared to NC, the *Nrf2* (Figure 2A), *CAT* (Figure 2B), *IgM* (Figure 2D), *LZM* (Figure 2E) and *AKP* (Figure 2F) mRNA expressions were decreased (*p* < 0.05) in HP. The mRNA expression of *Nrf2* was similar (*p* > 0.05) among NC, CT and EG, but was increased (*p* < 0.05) in CA and EC compared to NC and HP. The mRNA expression of *CAT* in CT, CA, EC and EG were lower (*p* < 0.05) than NC but were higher (*p* < 0.05) than HP. Compared to NC, the mRNA expression of *SOD* was similar (*p* > 0.05) in HP but was increased (*p* < 0.05) in CT, CA, EC and EG (Figure 2C), the mRNA expression of *IgM* was similar (*p* > 0.05) in CT but was increased (*p* < 0.05) in CA, EC and EG. There was no difference (*p* > 0.05) in the mRNA expression of *LZM* among CT, CA, EC and EG. The mRNA expression of *AKP* in CA, EC and EG were decreased (*p* < 0.05) compared to NC, but were increased (*p* < 0.05) compared to HP.

### 3.3 Growth performance of fish

Dietary treatments had minimal effects on growth performance of fish (Table 4). However, the FBW, WGR and SGR in G4 were increased (*p* < 0.05) compared to G3. All fish had similar (*p* > 0.05) SR, FI, FCR and ISI.

**Table 4:** Growth performance of fish fed experimental diets.

Items	Diets				
	G1	G2	G3	G4	G5
SR (%)	96.19 ± 0.95	96.19 ± 0.95	97.14 ± 1.65	96.19 ± 2.52	97.14 ± 1.65
FBW (g)	13.55 ± 0.25^ab^	13.61 ± 0.26^ab^	12.85 ± 0.17^b^	13.93 ± 0.40^a^	13.50 ± 0.10^ab^
WGR (%)	209.00 ± 5.35^ab^	210.87 ± 5.80^ab^	194.41 ± 4.14^b^	217.88 ± 9.07^a^	208.38 ± 0.49^ab^
SGR (%/d)	2.68 ± 0.04^ab^	2.70 ± 0.04^ab^	2.57 ± 0.03^b^	2.75 ± 0.07^a^	2.68 ± 0.01^ab^
FI (g/fish)	14.44 ± 0.12	14.67 ± 0.12	14.98 ± 0.63	15.17 ± 0.18	14.56 ± 0.42
FCR	1.58 ± 0.06	1.59 ± 0.06	1.77 ± 0.10	1.59 ± 0.05	1.60 ± 0.01
ISI (%)	0.87 ± 0.03	0.83 ± 0.10	0.92 ± 0.07	0.87 ± 0.04	0.92 ± 0.04

G1, basal diet; G2, basal diet supplemented with 1 g/kg of condensed tannins; G3, basal diet supplemented with 1 g/kg of catechin; G4, basal diet supplemented with 1 g/kg of epicatechin; G5, basal diet supplemented with 1 g/kg of epigallocatechin. SR, survival rate; FBW, final body weight; WGR, weight gain rate; SGR, specific growth rate; FI, feed intake; FCR, feed conversion rate; ISI, intestinesomatic index. Different superscript lowercase letters within a row indicate a significant difference among treatments from each other (*p* < 0.05).

### 3.4 Intestinal permeability and digestive enzyme activity of fish

The serum diamine oxidase and D-lactate concentrations, and the intestinal amylase activity of fish were not affected (*p* > 0.05) by dietary treatments (Table 5). Compared to G1, serum lipopolysaccharide concentrations in G2 and G3 were increased (*p* < 0.05), whereas intestinal trypsin and lipase activities were decreased (*p* < 0.05). Fish in G4 and G5 had similar (*p* > 0.05) lipopolysaccharide concentrations, and trypsin and lipase activities.

**Table 5:** Intestinal permeability and digestive enzyme activities of fish fed experimental diets.

Items	Diets				
	G1	G2	G3	G4	G5
Permeability					
Diamine oxidase (U/L)	21.55 ± 1.10	20.42 ± 1.48	20.87 ± 0.50	22.25 ± 0.93	20.98 ± 0.25
Lipopolysaccharide (pg/mL)	506.15 ± 8.52^b^	575.32 ± 11.15^a^	598.20 ± 5.96^a^	512.73 ± 8.81^b^	502.37 ± 8.89^b^
D-lactate (μmol/L)	2.52 ± 0.20	2.80 ± 0.05	2.68 ± 0.06	2.55 ± 0.08	2.88 ± 0.04
Digestive enzyme activities					
Trypsin (U/mg prot)	1433.46 ± 28.85^a^	1275.63 ± 10.05^b^	1259.31 ± 16.52^b^	1426.35 ± 17.60^a^	1468.20 ± 17.26^a^
Lipase (U/mg prot)	140.47 ± 5.97^a^	115.22 ± 2.35^b^	117.35 ± 9.98^b^	132.76 ± 8.15^ab^	140.78 ± 3.95^a^
Amylase (U/mg prot)	0.08 ± 0.01	0.06 ± 0.01	0.06 ± 0.01	0.07 ± 0.02	0.06 ± 0.01

G1, basal diet; G2, basal diet supplemented with 1 g/kg of condensed tannins; G3, basal diet supplemented with 1 g/kg of catechin; G4, basal diet supplemented with 1 g/kg of epicatechin; G5, basal diet supplemented with 1 g/kg of epigallocatechin. Different superscript lowercase letters within a row indicate a significant difference among treatments from each other (*p* < 0.05).

### 3.5 Intestinal histological appearance of fish

**Figure 3:**
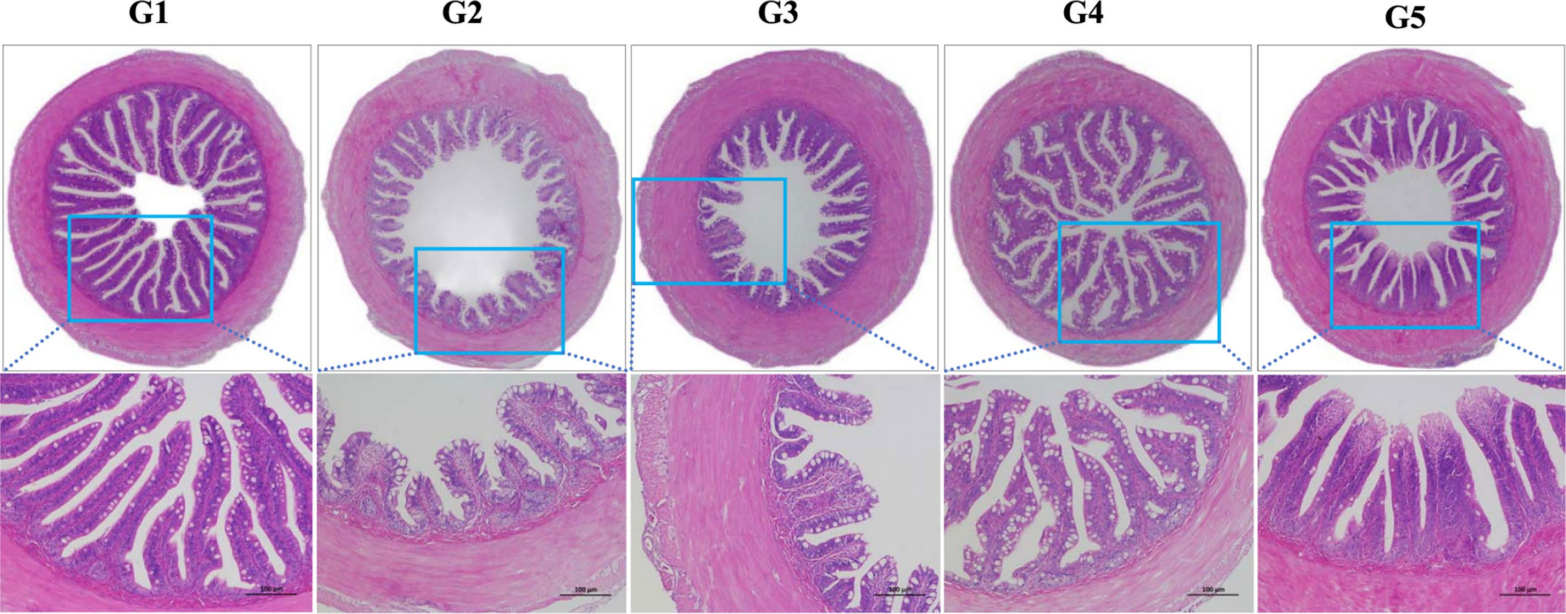
Intestinal histological appearance of *L. maculatus* fed experimental diets. Intestines were stained with hematoxylin and eosin (original magnification ×100). G1, basal diet; G2, basal diet supplemented with 1 g/kg of condensed tannins; G3, basal diet supplemented with 1 g/kg of catechin; G4, basal diet supplemented with 1 g/kg of epicatechin; G5, basal diet supplemented with 1 g/kg of epigallocatechin.

The intestinal histological appearance in G1 was normal in shape (Figure 3). The intestinal villus in G2 and G3 were damaged as reflected by the atrophic and irregular villus. In comparison, the intestinal villus in G4 and G5 were slightly damaged.

### 3.6 Intestinal antioxidant capacity of fish

The intestinal TAOC in G2 and G3 were increased (*p* < 0.05) compared to G1 and G4 (Table 6). The intestinal CAT activity was lower (*p* < 0.05) in G1 than that in other groups, but was similar among G2, G4 and G5. All fish had similar (*p* > 0.05) intestinal SOD activity. The intestinal MDA concentration in G1 was increased (*p* < 0.05) compared to other groups. Compared to G2, the intestinal MDA concentration was increased (*p* < 0.05) in G4 and G5.

**Table 6:** Intestinal antioxidant capacity of *L. maculatus* fed experimental diets.

Items	Diets				
	G1	G2	G3	G4	G5
TAOC (mmol/g prot)	0.10 ± 0.01^c^	0.20 ± 0.01^a^	0.17 ± 0.01^b^	0.13 ± 0.01^c^	0.16 ± 0.01^b^
CAT (U/mg prot)	3.10 ± 0.11^c^	3.86 ± 0.09^b^	4.50 ± 0.07^a^	3.70 ± 0.11^b^	3.82 ± 0.12^b^
SOD (U/mg prot)	36.88 ± 0.84	35.87 ± 0.73	35.50 ± 0.86	34.91 ± 0.57	36.49 ± 0.60
MDA (nmol/g prot)	14.64 ± 0.48^a^	7.88 ± 0.42^c^	8.72 ± 0.12^bc^	9.30 ± 0.22^b^	9.38 ± 0.08^b^

G1, basal diet; G2, basal diet supplemented with 1 g/kg of condensed tannins; G3, basal diet supplemented with 1 g/kg of catechin; G4, basal diet supplemented with 1 g/kg of epicatechin; G5, basal diet supplemented with 1 g/kg of epigallocatechin. TAOC, total antioxidant capacity; CAT, catalase; SOD, superoxide dismutase; MDA, malonaldehyde. Different superscript lowercase letters within a row indicate a significant difference among treatments from each other (*p* < 0.05).

### 3.7 Intestinal bacterial flora of fish

A total of 2,145,967 effective tags were obtained from all samples after filtering the low quality reads. All samples produced 11,448 operational taxonomic units (OTUs) with each sample averagely contained 763 OTUs after removing the singletons. The OTUs were assigned to 46 phyla, 100 class, 169 orders, 238 families, 376 genera and 187 species.

**Figure 4:**
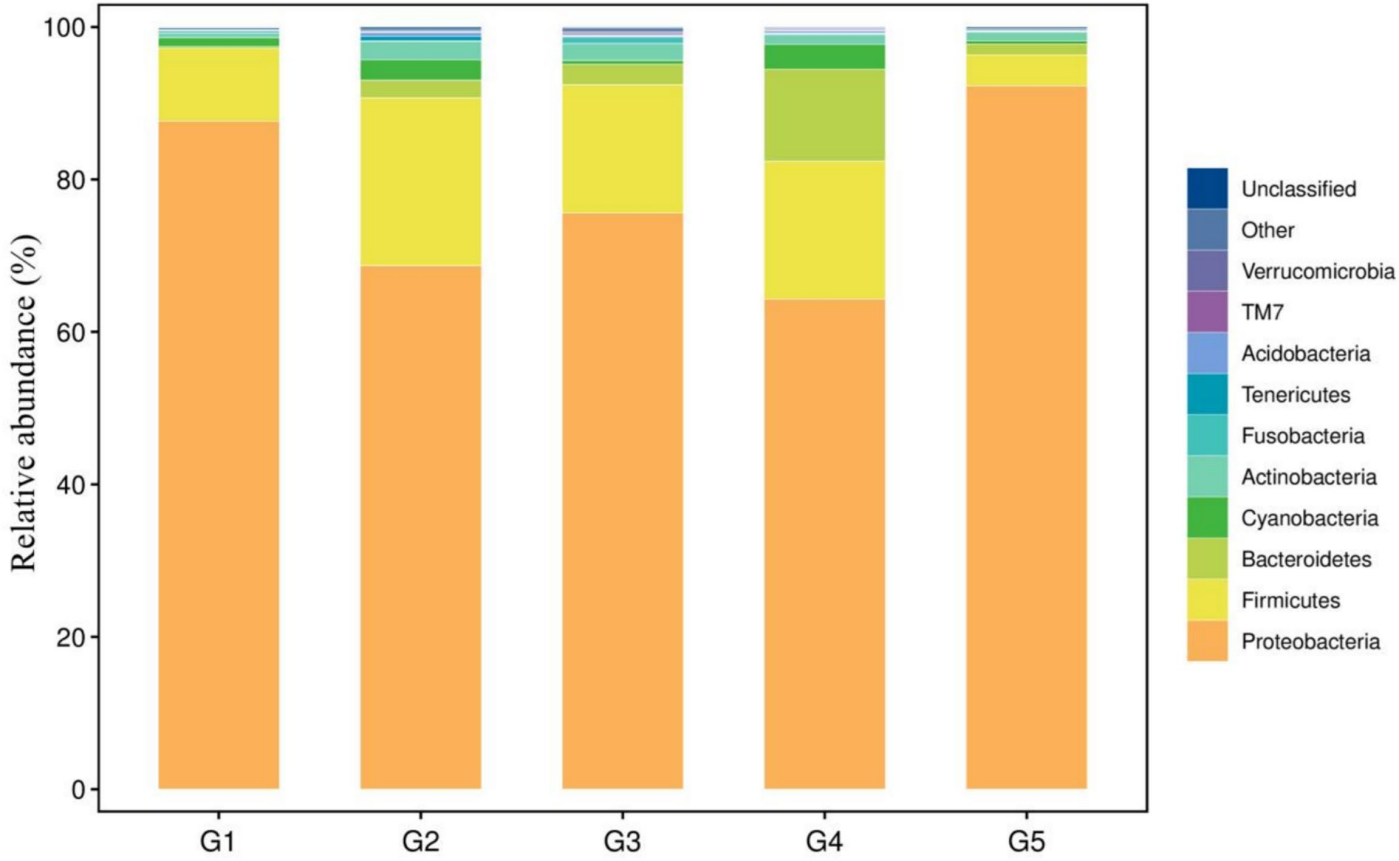
Taxonomic profile and relative abundance of the bacterial phyla. G1, basal diet; G2, basal diet supplemented with 1 g/kg of condensed tannins; G3, basal diet supplemented with 1 g/kg of catechin; G4, basal diet supplemented with 1 g/kg of epicatechin; G5, basal diet supplemented with 1 g/kg of epigallocatechin.

**Figure 5:**
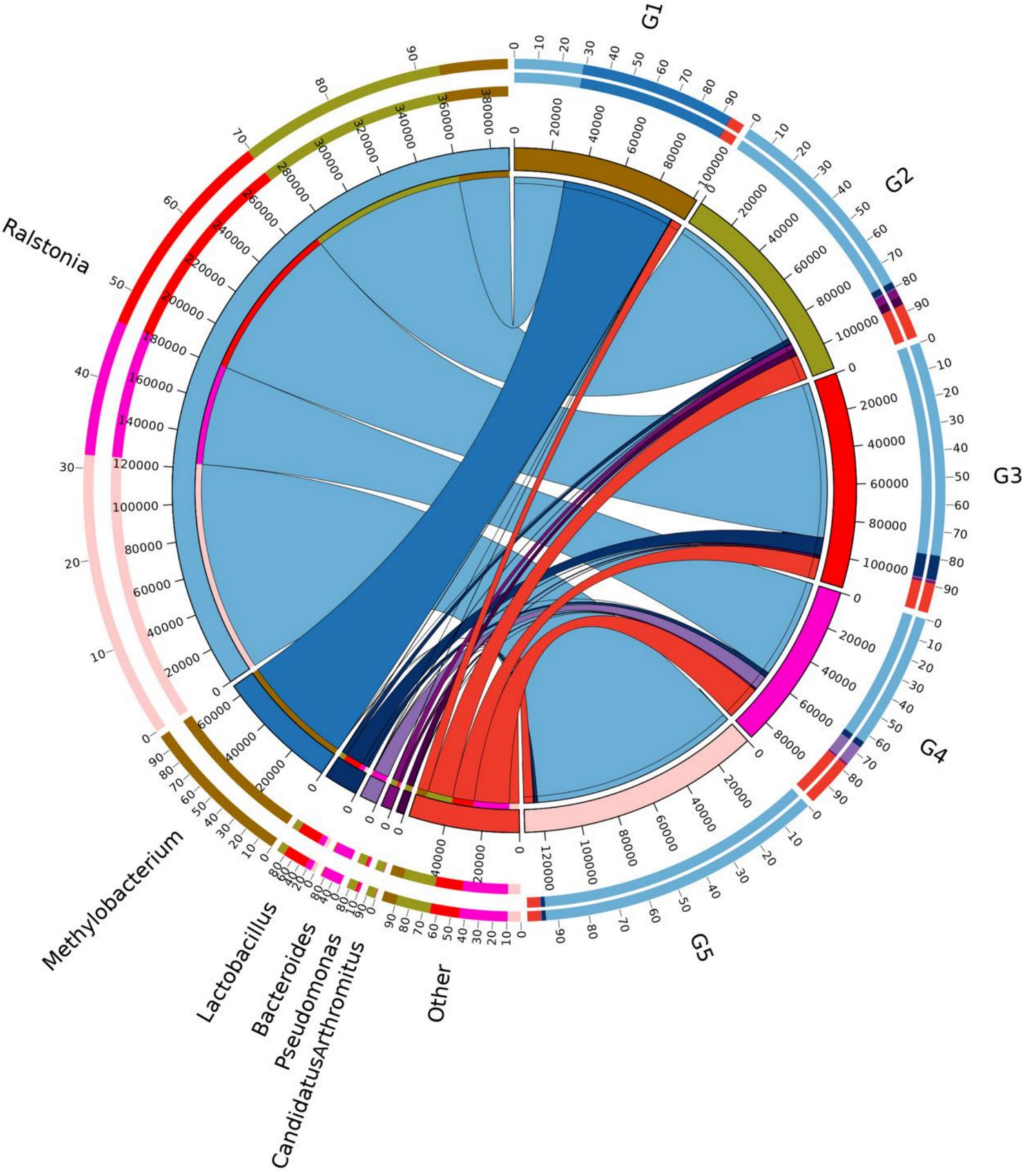
Relationship between diets and relatively abundant bacterial genera. G1, basal diet; G2, basal diet supplemented with 1 g/kg of condensed tannins; G3, basal diet supplemented with 1 g/kg of catechin; G4, basal diet supplemented with 1 g/kg of epicatechin; G5, basal diet supplemented with 1 g/kg of epigallocatechin.

The top 10 dominant bacterial phyla irrespective of the dietary treatment were the Proteobacteria, Firmicutes, Bacteroidetes, Cyanobacteria, Actinobacteria, Fusobacteria, Tenericutes, Acidobacteria, TM7 and Verrucomicrobia (Figure 4). The relationship between diets and the top 6 bacterial genera (*Ralstonia*, *Methylobacterium*, *Lactobacillus*, *Bacteroides*, *Pseudomonas* and *Clostridium*) was shown in Figure 5. Compared to G1, the relative abundance of *Ralstonia* was increased (*p* < 0.05) but the relative abundance of *Methylobacterium* was decreased (*p* < 0.05) in G2, G3, G4 and G5.

**Figure 6:**
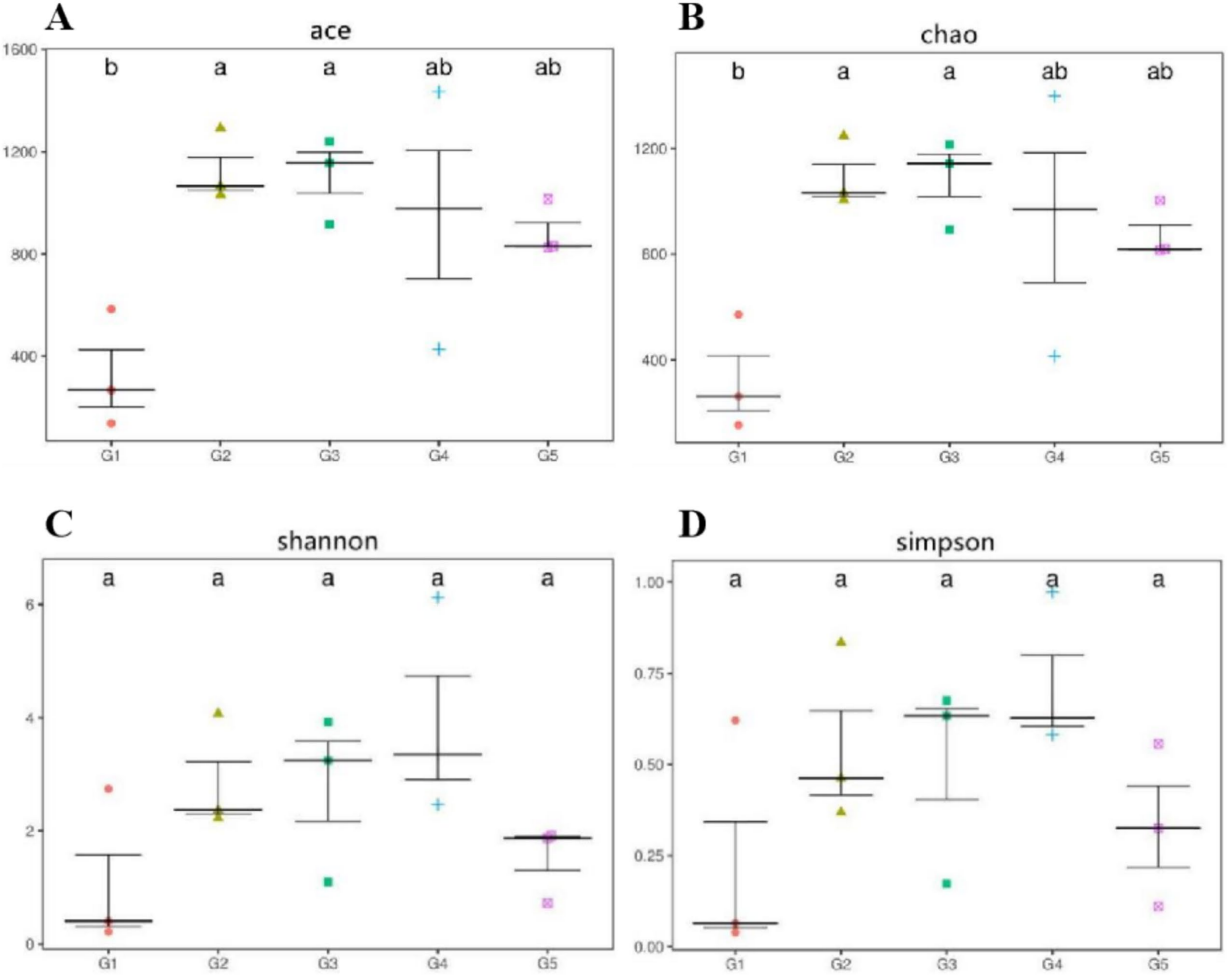
Alpha diversity measurements of bacterial communities at the genus level. Alpha diversity measured as follows: ace and chao **(A,B)**, richness estimators to estimate the total number of OTUs present in a community; shannon and simpson **(C,D)**, microbial index of diversity. G1, basal diet; G2, basal diet supplemented with 1 g/kg of condensed tannins; G3, basal diet supplemented with 1 g/kg of catechin; G4, basal diet supplemented with 1 g/kg of epicatechin; G5, basal diet supplemented with 1 g/kg of epigallocatechin.

**Figure 7:**
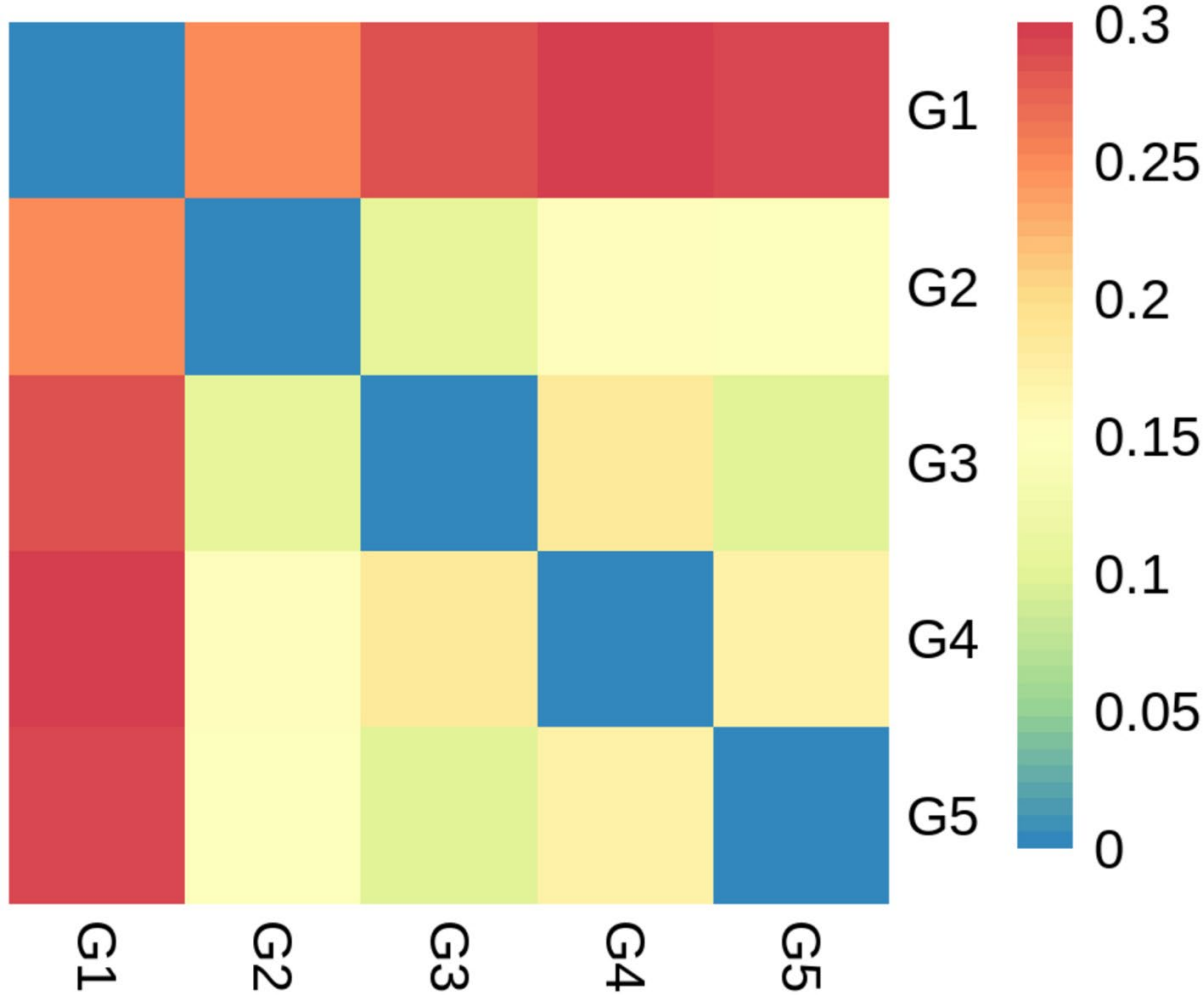
Beta diversity based on bacterial community membership metric with the Weighted-Unifrac. G1, basal diet; G2, basal diet supplemented with 1 g/kg of condensed tannins; G3, basal diet supplemented with 1 g/kg of catechin; G4, basal diet supplemented with 1 g/kg of epicatechin; G5, basal diet supplemented with 1 g/kg of epigallocatechin.

Alpha diversity measurements showed that the ace and chao were increased (*p* < 0.05) in G2 and G3 compared to G1 (Figures 6A,B). However, all groups had similar (*p* > 0.05) shannon and simpson indexes (Figures 6C,D). Weighted-Unifrac beta diversity that compared the similarity in the intestinal bacterial community composition was significantly different (*p* < 0.05) among groups (Figure 7).

**Figure 8:**
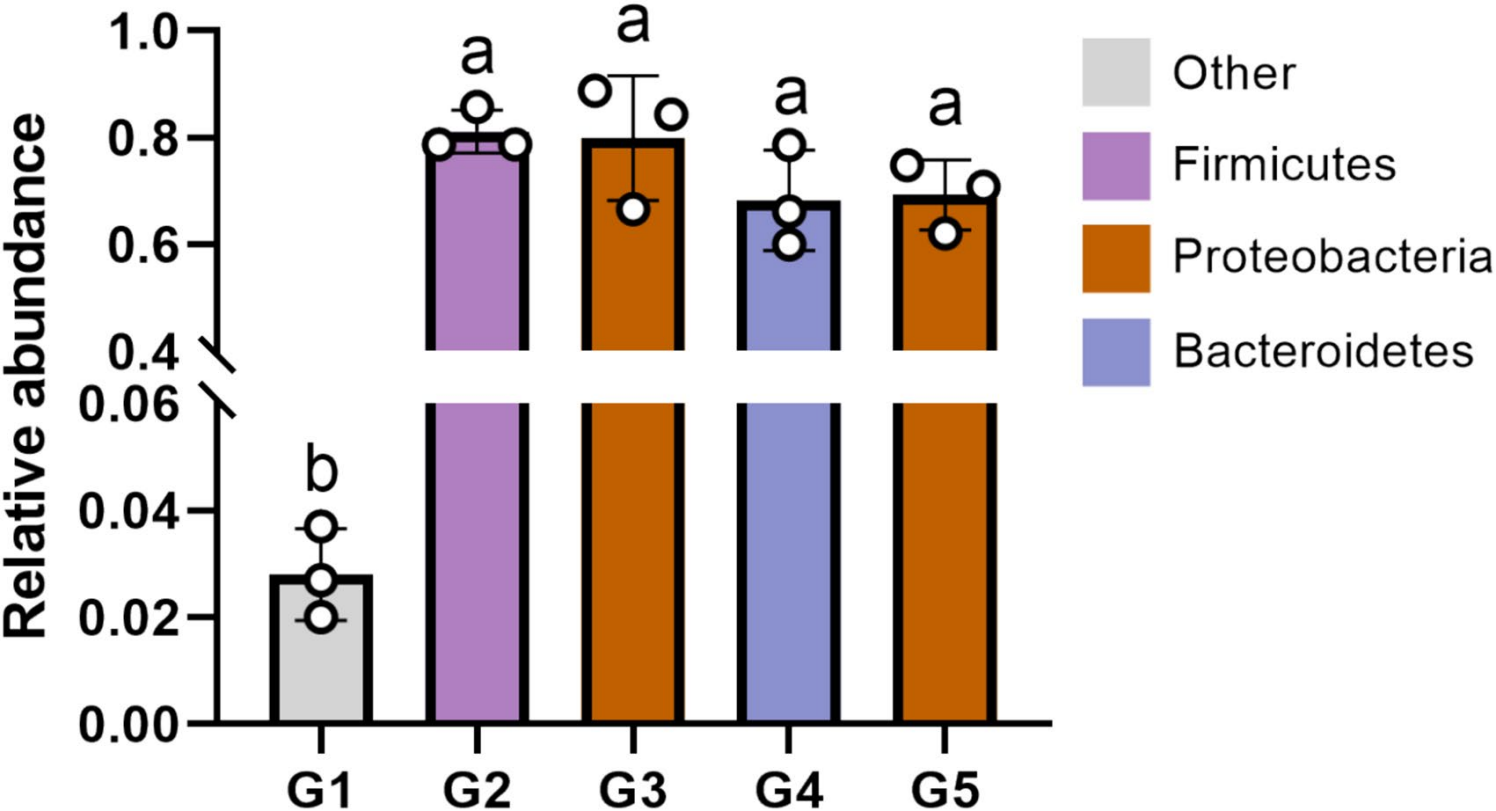
The phenotypic contribution of bacterial phyla to oxidative stress tolerant using Bugbase phenotypic classification prediction analysis. G1, basal diet; G2, basal diet supplemented with 1 g/kg of condensed tannins; G3, basal diet supplemented with 1 g/kg of catechin; G4, basal diet supplemented with 1 g/kg of epicatechin; G5, basal diet supplemented with 1 g/kg of epigallocatechin. Different lowercase letters above the bars denote significant differences among treatments from each other (*p* < 0.05).

Among the identified phyla, the Firmicutes, Proteobacteria and Bacteroidetes were the primary phenotypic bacteria contributing to oxidative stress tolerant in G2, G3, G4 and G5 (Figure 8). Other bacterial phyla contributed to the oxidative stress tolerant in G1. Compared to G1, the oxidative stress tolerance of the intestinal bacteria abundance were increased (*p* < 0.05) in other groups.

## 4 Discussion

In this study, the decreased cell viability along with the weakened hydroxyl radical scavenging ability and anti-superoxide anion activity may account for the reduced antioxidant activity and immunity of cells induced by HP, because the accumulation of ROS can damage the antioxidant and defense system (34, 35). CT are polyphenolic compounds that possess strong antioxidant and immune-promoting function and have significant bioactivity to repair oxidative damage (2, 17, 36). In this study, both of CT and CAs were observed to promote cell viability, antioxidant activity and immunity, indicating CT and CAs alleviated HP-induced cell injury. This is consistent with previous reports that CT and CAs increased cell viability (37–40). Based on the HP-induced cell oxidative stress model, CT and CAs had similar cell hydroxyl radical scavenging ability and anti-superoxide anion activity, whereas EC was observed to have superior ability to enhance cell viability than CT or other monomers (CA and EG). This may due to the better activation of Nrf2 signaling pathway by EC as reflected by the increased gene expression of *Nrf2*, because activation of the Nrf2 signaling pathway was reported to significantly increase cardiomyocyte viability, reduced ROS formation and enhanced antioxidant enzyme activity in a Type 2 diabetic rat model (41). The increased gene expression of *Nrf2* in this study may suggest CT and CAs improved cell antioxidant capacity by activating the Nrf2 signaling pathway (42). Our previous studies also reported that CT enhanced oxidation resistance of *L. maculatus* by upregulating the mRNA levels of *Nrf2* and antioxidase (6). Similarly, CT were reported to significantly reinforce the immunity of oxidation-injured *L. maculatus* via increasing immune indices of AKP, LZM and IgM (36).

Although the antioxidant and immune-promoting activities of CT have been well documented (2, 43), information of such effects of CAs in fish is scarce. Based on the HP stress model, this study is the first to compare the effects of CT and CAs on repairing injured intestinal cells of fish. Although CAs displayed similar cell anti-superoxide anion activity to that of CT, discrepancy was observed among CAs examined in cell viability. For instance, cell viability was higher in cells treated with EC, similar in CA, but lower in EG as compared with CT. However, this discrepancy is inconsistent with the observation in antioxidant and immune indices, suggesting the discrepancy in cell viability is unlikely due to their individual differences in antioxidant and immune capacities. Commonly, ROS can induce cell senescence and death (44), and therefore the cell viability depends on its ability to remove ROS. Liu and Tian (45) reported that EC had stronger free radical scavenging capacity than CA. Tu et al. (46) indicated that the scavenging ROS ability of EC was greater than that of EG. Piao et al. (47) also found that EC had superior protective effect on lead-exposed HepG2 cell viability than EG. In this study, the discrepancy in cell viability of CT and CAs may attribute to their individual differences in scavenging ROS ability.

TAOC refers to the total antioxidant capacity composed of various antioxidants and antioxidant enzymes. The higher activity and gene expression of antioxidases observed in CA and EC indicated that these two monomers possessed stronger antioxidant capacity than CT and EG. This combined the observation that CAs had similar CAT and GPx activities to that of CT suggested the monomers of CT could be considered as equivalent or superior antioxidants compared to CT. In this study, CA and EC had similar antioxidant activity, likely due to their comparable antioxidant potentials (48). Compared with control and other supplements, CA and EC significantly up-regulated the mRNA expression of *Nrf2*. This was similar to previous reports that CA and EC were considered as the Nrf2 activator (49, 50).

This study showed that CT and CAs partly or fully repaired immune damage. Previous studies reported that tannins inhibit pro-inflammatory cytokines secretion and therefore exert anti-inflammatory activity by inhibiting the nuclear factor kappa-B (NF-κB) signaling (6, 51, 52). In this study, the concentration and mRNA level of IgM in cells treated with CA, EC and EG were even significantly surpassed those of NC and CT. The observation that CT and CAs regulate immune indices to a different extent was likely attribute to the differences in their regulation mechanism in immune function. CT have been reported to inhibit the production of pro-inflammatory factor during cellular inflammatory response (53) or possess anti-inflammatory activity via inhibition of NF-κB and mitogen-activated protein kinase (MAPK) signaling pathways (54). CA and EC were reported to influence the immune response by modulating NF-κB activation (55) or decreasing cell permeability and protecting mitochondrion (56). It was documented that EG (tea catechin epigallocatechin gallate) inhibited NF-κB-mediated transcriptional activation by covalent modification which was thought to have contribution to anti-inflammatory (57).

Although the feeding value of CT for *L. maculatus* has been well evaluated (6, 17, 31, 58, 59) little information is available about CAs. This is the first study to compare the effects of CT and CAs on growth performance and intestinal health of *L. maculatus.* The similar growth performance across dietary treatments suggested that 1 g/kg of CT and CAs did not significantly affect growth performance of fish. This is consistent with our previous reports that dietary CT at 1 g/kg did not alter growth performance of *L. maculatus* (17, 36). Similar observation for CA supplementation were also documented in grass carp (60), meat duck (61), weaning pig (62) and broiler (63). However, other reports documented that CT and CAs have positive (64–66) or negative (67–69) effects on the growth performance of animals. Probably it is regarded to the variation of dietary dose, animal species and feeding duration among studies.

The concentration of blood lipopolysaccharide commonly reflect the permeability or intestinal injury degree in fish, because lipopolysaccharide could get into the bloodstream via the damaged intestine (70). In this study, the increased serum lipopolysaccharide concentrations in fish fed CT and CA as compared to that of control indicated that these compounds induced intestinal injury of *L. maculatus*, which is supported by the observation of intestinal histological appearance. Chen et al. (59) found that dietary CT at 1 g/kg did not affect intestinal permeability, whereas 2 g/kg of CT increased the concentration of serum lipopolysaccharide and thus increased intestinal permeability of *L. maculatus* by destroying intestinal tight junction structure and interfering intestinal bacteria and metabolites. Difference between this study and Chen et al. (59) may due to differences in CT dose, because the actual concentrations of CT in this study (1.2 g/kg) was greater than that (1 g/kg) of Chen et al. (59). Peng et al. (58) documented that inclusion of 1 g/kg CT in *L. maculatus* diets reduced the intestinal villus length. Li (71) also reported that CT induced intestinal cell apoptosis and damaged the intestinal tight junction structure and integrity of grass crap. However, some previous studies suggested that CT or CA played a role in protecting intestine or repairing intestinal injury (5, 72, 73). Discrepancy among studies may be attributed to animal species and dietary dose. As for digestive enzyme activities, the decreased intestinal trypsin and lipase activities in fish fed CT and CA indicated that they may interfere with the digestion and absorption of protein and lipid. Qiu et al. (74) reported that dietary CT at 1 g/kg significantly inhibited the intestinal trypsin and lipase activities of *Litopenaeus vannamei*. Peng et al. (44) found dietary CT inhibited the digestion of protein and lipase due to the destruction of intestine by CT. Fei (75) documented that oolong tea CA significantly inhibited digestive enzymes activities *in vitro*, owing to the combination of CA and enzymes forming a relatively stable complex that decreased enzyme activity.

Antioxidant studies in fish often focus on blood and liver, whereas information in terms of intestinal antioxidant capacity is limited. Because the intestine is constantly exposed to various exogenetic substances, it is also very easy to produce various oxidative stress. To our best knowledge, this is the first study to evaluate the effects of CT and CAs on intestinal antioxidant capacity of fish. Overall, dietary inclusion of 1 g/kg of CT or CAs improved antioxidant capacity of *L. maculatus*. Although the catalase and superoxide dismutase activities as well as MDA concentrations among treated diets were similar, CT and CA showed superior antioxidant capacity than other diets as reflected by the increased intestinal TAOC. Previous studies reported that both CT and CA were strong antioxidants for livestock, poultry and aquatic animals (6, 60, 66, 72). Zhu (76) indicated EC relieved lead-intoxication of mice by activating the Nrf2 signaling pathway and improving antioxidant enzyme activities. EG was also regarded as an activator of the Nrf2 signaling pathway (77), which showed superior antioxidant and free radical scavenging activities *in vivo* and *in vitro* (78).

This study showed that the dominant bacterial phyla in the intestine of *L. maculatus* were Proteobacteria and Firmicutes, which are regarded as the major bacterial phyla in the gut of fish (79). This observation is in agreement with previous reports by Peng et al. (18) and Chen et al. (59). At genus level, dietary CT and CAs increased the relative abundance of *Ralstonia* and decreased the abundance of *Methylobacterium*. As the Gram-negative species, intestinal *Ralstonia* was reported to augment glucose intolerance (80). Thus, the increased intestinal *Ralstonia* abundance may due to the inhibition of blood glucose by inclusion of CT and CAs in diets, because CT and CAs were proved to decrease blood glucose in fish (81, 82). The *Methylobacterium* were considered as a class of potential pathogenic bacteria in fish gut (83). In this study, the decreased *Methylobacterium* abundance suggested that dietary CT and CAs at 1 g/kg inhibited growth of intestinal pathogenic bacteria *Methylobacterium*, which would be beneficial for promoting intestinal health of the fish. The analysis of bacterial diversity suggested that inclusion of CT and CAs in diets altered the structure of bacterial communities. For instance, the increased alpha diversity measurements of ace and chao indicated that CT and CA significantly increased the total number of OTUs in the bacterial communities. This is consistent with the observation by Peng et al. (31) that dietary CT increased the bacterial diversity measurements of ace and chao in the intestine of *L. maculatus.* Similarly, CAs (the main component of tea polyphenols) have been shown to increase the diversity of intestinal flora in mice (84). In this study, the Firmicutes, Proteobacteria and Bacteroidetes were not only the primary bacterial phyla in the intestine of fish, but also the identified phyla contributed to the oxidative stress tolerance. The increased relative abundance of these phyla suggested that supplementation of CT and CAs enhanced the intestinal antioxidant capacity of fish. This is agreement with the observation in the evaluation of intestinal antioxidant capacity. Despite the relative abundance of phyla contributed to the oxidative stress tolerant among treated diets were similar, their individual contributed bacterial phyla were different. In other words, the Firmicutes and Bacteroidetes mainly contributed to the antioxidant function of CT and EC, while the Proteobacteria contributed to the antioxidant function of CA and EG. Currently, an increasing number of literatures reported that the intestinal microbiota is a key factor in the therapeutic effects of (poly)phenols (85–88). This study provides a new perspective for further exploration of the antioxidant mechanism of CT and CAs in the aspect of intestinal microbiota.

## 5 Conclusion

In conclusion, *in vitro* study showed that CT and CAs exhibited similar effects in alleviating hydrogen peroxide-induced injury of intestinal cells by activating Nrf2 factor and improving antioxidant and immune capacity. EC was observed to have superior ability to enhance cell viability than CT or other monomers. Animal feeding trial indicates that inclusion of CT and CAs in *L. maculatus* diets did not affect growth performance, but enhanced intestinal antioxidant ability and increased relative abundances of bacterial phyla Firmicutes, Proteobacteria and Bacteroidetes to oxidative stress tolerance. With a dose of 1 g/kg CT and CA significantly promoted total antioxidant capacity of intestine, but slightly induced intestinal damage of fish mainly due to the increased intestinal permeability and bacterial diversity along with inhibited digestion. Throughout the text, without affecting the growth performance of fish, CA and EC show similar or superior antioxidant activity than CT. The findings from this study will give a reference for the application of CT in aquaculture and provide new insights by dissecting the roles of condensed tannins in promoting antioxidant and intestinal health of fish using catechins monomers.

## Data Availability

The 16S sequencing raw data was deposited in the NCBI SRA database with the BioProject accession number PRJNA1032849. All other data used in this study are available from the corresponding author upon reasonable request.
